# S1PR-1/5 modulator RP-101074 shows beneficial effects in a model of central nervous system degeneration

**DOI:** 10.3389/fimmu.2023.1234984

**Published:** 2023-08-09

**Authors:** Mustafa Sindi, Christina Hecker, Andrea Issberner, Tobias Ruck, Sven G. Meuth, Philipp Albrecht, Michael Dietrich

**Affiliations:** ^1^ Department of Neurology, Medical Faculty of the Heinrich-Heine University, Düsseldorf, Germany; ^2^ Department of Neurology, Maria Hilf Clinics, Mönchengladbach, Germany

**Keywords:** S1PR-1, S1PR-5, RP-101074, neuroprotection, multiple sclerosis, microglia, CX3CR1

## Abstract

**Introduction:**

In multiple sclerosis (MS), chronic disability primarily stems from axonal and neuronal degeneration, a condition resistant to conventional immunosuppressive or immunomodulatory treatments. Recent research has indicated that selective sphingosine-1-phosphate receptor S1PR-1 and -5 modulators yield positive effects in progressive MS and mechanistic models of inflammation-driven neurodegeneration and demyelination.

**Methods:**

In this study, the S1PR-1/-5 modulator RP-101074 was evaluated as a surrogate for ozanimod in the non-inflammatory, primary degenerative animal model of light-induced photoreceptor loss (LI-PRL) in CX3CR1-GFP mice to assess potential neuroprotective effects, independent of its immunomodulatory mechanism of action.

**Results:**

Prophylactic administration of RP-101074 demonstrated protective effects in the preclinical, non-inflammatory LI-PRL animal model, following a bell-shaped dose-response curve. RP-101074 treatment also revealed activity-modulating effects on myeloid cells, specifically, CX3CR1+ cells, significantly reducing the marked infiltration occurring one week post-irradiation. Treatment with RP-101074 produced beneficial outcomes on both retinal layer thickness and visual function as evidenced by optical coherence tomography (OCT) and optomotor response (OMR) measurements, respectively. Additionally, the myelination status and the quantity of neural stem cells in the optic nerve suggest that RP-101074 may play a role in the activation and/or recruitment of neural stem cells and oligodendrocyte progenitor cells, respectively.

**Conclusion/Discussion:**

The data from our study suggest that RP-101074 may have a broader role in MS treatment beyond immunomodulation, potentially offering a novel approach to mitigate neurodegeneration, a core contributor to chronic disability in MS.

## 1 Introduction

Multiple Sclerosis (MS) is an inflammatory autoimmune disorder characterized by demyelination, oligodendrocyte loss, subsequent axonal damage, and eventual neuronal degeneration within the central nervous system (1). This degeneration primarily accounts for the persistent clinical disability in MS patients, which remains unresponsive to conventional immunosuppressants and most immunomodulatory therapies. MS-related pathological alterations are typically marked by the infiltration of lymphocytes and macrophages into the CNS parenchyma, instigating the upregulation of adhesion molecules and promoting immune cell recruitment via inflammatory cytokines. This cascade results in glial and neuronal injury through a not yet fully elucidated mechanism. Increased oxidative stress has been hypothesized to play a significant role in neuronal damage (2), as activated immune cells may release reactive oxygen or nitrogen species.

Ozanimod, siponimod, and ponesimod are oral sphingosine-1-phosphate (S1P) receptor modulators with varying selectivities for different S1P receptor sub-types and have been the focus of extensive research in recent years (3–5). Ozanimod and siponimod selectively target S1P receptor sub-types 1 and 5, while ponesimod is selective for S1P receptor sub-type 1. These selective S1P receptor modulators have demonstrated promising efficacy in the treatment of relapsing-remitting multiple sclerosis (RRMS) (6) and secondary progressive multiple sclerosis (SPMS) (5). Presently, research is being conducted to understand the brain-protecting effects of these selective S1P receptor modulators during the later stages of multiple sclerosis. This could potentially provide new treatment alternatives for patients suffering from progressive forms of the disease. Within this comprehensive framework for, we are increasingly incorporating state-of-the-art technologies to enhance our monitoring capabilities. Optical coherence tomography (OCT), for instance, is proving to be instrumental due to its non-invasive nature and ability to provide high-resolution, detailed images of the retina within the eye, thereby enabling a more precise understanding of disease progression and response to treatment. Previous research has demonstrated a notable reduction in the thickness of the retinal nerve fiber layer (RNFL) and the ganglion cell layer-inner plexiform layer complex (GCIP) in MS patients as a consequence of optic neuritis, as well as in the eyes of MS patients without optic (7–9). Retinal degeneration in MS patients not only serves as a morphological correlate of functional visual deficits but also reflects overall disability, such as that assessed by the Expanded Disability Status Scale (EDSS) (7, 9, 10), and brain atrophy measured by MRI (10–12). This renders OCT an ideal tool for visualizing neurodegeneration, neuroprotection, and neuro-repair processes in both clinical and preclinical settings.

Our study revolves around the murine light-induced photoreceptor loss (LI-PRL) model, which represents a primarily non-inflammatory neurodegenerative condition. The aim was to assess the effectiveness of the compound RP-101074 in the chronic stages of degenerative CNS disease. This also applies to the chronic phases of MS, where lymphocyte and monocyte infiltration and inflammation play a less significant role in driving pathology, while neurodegeneration and activation of microglia emerge as the dominant characteristics. Unfortunately, there is not an ideal animal model to perfectly mimic this condition. However, the LI-PRL model induces CNS damage and subsequently inflammation and degeneration in the retina that is independent of T cell activity, thus making it a valuable model for our investigation. Although LI-PRL provokes retinal inflammation, it is not chronic as in the experimental autoimmune encephalomyelitis (EAE) model, but rather undergoes rapid remission. This period of remission of inflammation presents a critical window for us to observe neurodegeneration and to evaluate the potential neuroprotective effects of the compound. This model has been instrumental in our previous investigations, where we explored the neuroprotective effects of dimethyl fumarate, an immunomodulatory substance with potential neuroprotective capabilities (13). Building upon this previous work, we further extended our research to examine the protective capabilities of various doses of the S1PR-1/-5 modulator and ozanimod surrogate RP-101074 on retinal degeneration and visual function within the same LI-PRL model. The objective was to investigate if RP-101074 exhibits neuroprotective properties that could signify potential benefits in disorders characterized by primary neurodegeneration.

## 2 Material and methods

### 2.1 Animals

Six-week-old, female C57Bl/6J mice and CX3CR1-GFP transgenic mice were utilized in this study. For the CX3CR1-GFP mice, B6.129P2(Cg)-Cx3cr1tm1Litt/J mice (CX3CR1-GFP) were originally obtained from “The Jackson Laboratory” and fully backcrossed into the C57BL/6J strain at the animal facility at Heinrich Heine University in Düsseldorf, Germany. Additionally, we ensured the absence of the RD8 mutation, which is associated with hereditary macular degeneration. C57Bl/6J mice were purchased from Janvier Labs, France. In LWI-LI-PRL, n=5 animals (10 eyes) per group were used in two independently performed experiments. In HHI-LI-PRL, n=6 animals (12 eyes) per group were used in two independently performed experiments.

### 2.2 Light-induced photoreceptor loss and treatment

Prior to irradiation using an LED light source (cold temperature), the pupils were dilated with a combination of 0.5% Tropicamide and 2.5% Phenylephrine. To prevent dehydration and cataract formation during irradiation, the eyes were treated with eye gel. Both eyes were exposed to irradiation for 45 minutes at maximum intensity and with the fully opened shutter. The distance between the light source and the eye was set at 10 mm for weak degenerative conditions (Low Intensity, LWI) and 5 mm for strong degenerative conditions (High Intensity, HHI).

Mice were treated daily, from the day of irradiation until the end of the experiment after either 5 or 6 weeks, with RP-101074 at doses of 0.1 mg/kg, 0.3 mg/kg, 1 mg/kg, and 5 mg/kg body weight (BW), administered via oral gavage.The vehicle control group was subjected to the same procedures as the treatment group, including anesthesia and eye preparation, but without irradiation. Animals of the vehicle group were administered with a vehicle solution of PBS/DMSO (1%).

### 2.3 OCT measurements

Periodic OCT measurements were conducted using the Spectralis HRA + OCT device (Heidelberg Engineering, Germany) with several adaptations for rodents, as previously described (14). Segmentation of retinal volume scans was performed using the Heidelberg Eye Explorer software, with manual control for segmentation errors. The volume of the para-papillary region was assessed using the ETDRS grid, excluding the center with the disc, as published previously (15). Inner retinal layer (IRL) thickness, outer retinal layer (ORL) thickness, and total retinal thickness (TRT) were examined at defined intervals after irradiation and compared with baseline measurements (IRL: NFL, GCL, and IPL layers; ORL: INL, OPL, ONL, ELM, IS/OS, RPE, Choroid; TRT: IRL + ORL).

### 2.4 Confocal scanning laser ophthalmoscopy measurements

**Figure 1:**
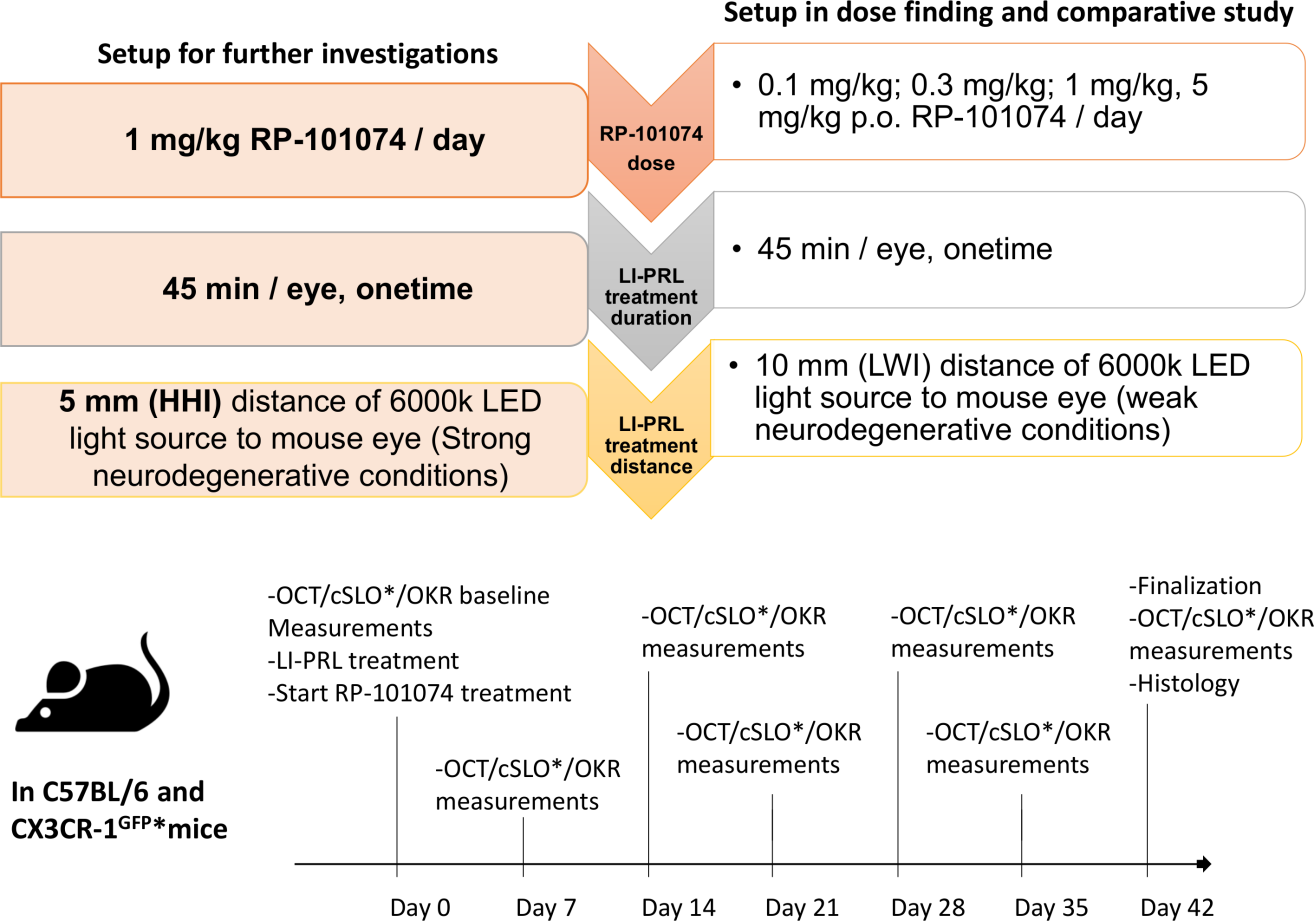
Experimental design, treatment timeline and setup conditions. Experimental setup of RP-101074 dose-response and further investigations. The dose-response study was conducted for 5 weeks, utilizing 0.1, 0.3, 1, and 5 mg/kg/day RP-101074, with light-induced photoreceptor loss (LI-PRL) administered from a 10mm distance (weak neurodegenerative conditions, LWI). For strong neurodegenerative conditions (HHI), the study was conducted for 6 weeks with 1 mg/kg/day RP-101074 and 5mm distance LI-PRL. Readouts were performed weekly, with euthanization and histological assessment post-study. An asterisk (*) denotes the CX3CR-1 GFP mice subset, which also underwent additional confocal scanning laser ophthalmoscopy (cSLO).

**Figure 2:**
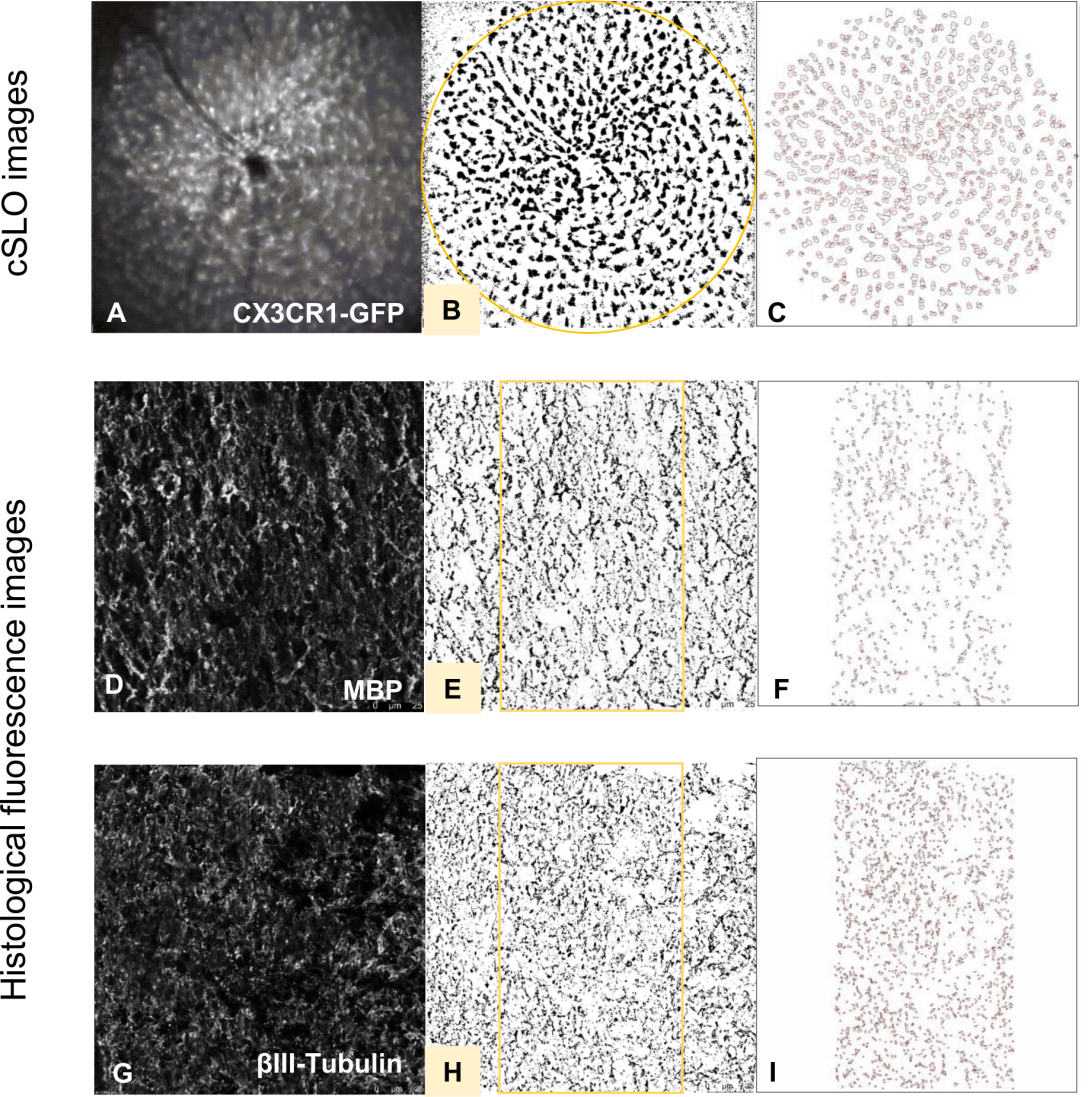
Histological evaluation process of fluorescence and cSLO images. cSLO: Initially, **(A)** CX3CR1-GFP labeled cells in the retina were captured by cSLO. Then, a **(B)** calibrated oval mask section was placed around the optic disc, followed by a **(C)** automated cell count using Fiji-ImageJ2 software, as per the protocol developed by Frenger et al. (16). Fluorescence images: Fluorescence-stained tissues with different markers were captured and processed **(D, G)**. Then, a **(E, H)** calibrated rectangle mask section was applied in the middle of the optic nerve, followed by a **(F, I)** automated total area measurement using Fiji-ImageJ2 software.

CSLO was performed periodically (**Figure 1**) using the 480 nm laser and the fluorescence angiography filter of the Spectralis® device (as described in 2.3) for imaging retinal microglia in CX3CR1-GFP transgenic mice. The number of GFP-positive cells in a predefined oval mask around the optic disc was counted using a modified automated algorithm developed by 16 (**Figure 2**). By employing the CX3CR1-GFP mouse line, the activity of CX3CR1+ myeloid cells was longitudinally analyzed in the retina to draw conclusions about the effects of RP-101074 treatment on microglial cells.

### 2.5 Anesthesia

For the photoreceptor loss (PRL) treatment and Optical Coherence Tomography (OCT)/confocal Scanning Laser Ophthalmoscopy (cSLO) measurements, the animals were anesthetized using isoflurane (Vaporizer from Harvard Apparatus Anesthetic Vaporizors; Isofluran from Piramal critical care). Specifically, induction was carried out at 3.5% isoflurane, followed by maintenance at 2% isoflurane, with a flow rate of 0.6 L/minute of oxygen.

### 2.6 Optomotor response measurements

OMR measurements were performed periodically (**Figure 1**) using the OptoMotry® device from Cerebral Mechanics, in parallel with OCT and cSLO measurements. Spatial frequency was monitored as a parameter for visual function. The spatial frequency threshold was determined by randomly changing the spatial frequency to identify the threshold at which the mouse could track, as previously described (15, 17).

### 2.7 Histology

Mice were anesthetized with Ketamine/Xylazine, euthanized using an overdose of isoflurane (Piramal critical care), and cardiac perfusion was performed using phosphate-buffered saline (Gibco, Carlsbad, USA). The optic nerves were then isolated and fixated in 4% paraformaldehyde (Carl Roth, Karlsruhe, Germany) overnight. After fixation, the optic nerves were subjected to a sucrose gradient for dehydration and subsequently embedded in O.C.T. compound (Sakura™ Finetek, Alphen aan den Rijn, The Netherlands). Longitudinal sections of five micrometers were cut and prepared for fluorescence staining. Longitudinal sections of the optic nerves were used for quantifying neural stem cells (Sox2, (Clone E-4) Santa Cruz Biotechnology, 1:200), assessing the state of myelination (MBP (Clone 12), Merck Millipore, 1:200), and evaluating neuronal survival (βIII-tubulin (Clone TUJ1), Biolegend, 1:200) using Leica HyD detector attached to a Leica DMi8 confocal microscope (63x objective lens magnification). Cy5 rat anti-mouse, Cy5 rabbit anti-mouse (1:500, Millipore) and Alexa Fluor™ 488 rabbit anti-mouse (1:500, Invitrogen) were used as secondary antibodies. The numbers of cells stained with Sox2 were analyzed using ImageJ software, applied by blinded raters, and expressed as a ratio to DAPI staining. The overall signal for MBP and βIII-tubulin (positive total area in the red channel) was analyzed using ImageJ software as shown in **Figure 2**.

### 2.8 Statistics

Statistical analysis was performed using Prism (version 9, GraphPad® Software, Inc.) and IBM SPSS Statistics (version 20, IBM Corporation, USA). Total and percent changes of the acquired retinal parameters (OCT, OMR, and cSLO) were analyzed using generalized estimation equation (GEE) models, accounting for within-subject inter-eye correlations, to test for differences between the two groups. For non-paired data, group means analyses were compared using a one-way ANOVA with the Dunnett *post hoc* test, utilizing one optic nerve per animal for the histological investigations.

## 3 Results

### 3.1 Retinal structural and visual function analysis after LI-LWI-PRL treatment (weak neurodegenerative conditions)

To mimic the range of damage severity seen in multiple sclerosis, we conducted a longitudinal investigation on C57BL/6J mice, applying two levels of light-induced damage. The rationale behind this approach was to simulate both mild and severe forms of retinal degeneration that occur in the disease course. Therefore, we weekly evaluated the potential of RP-101074 in protecting against retinal degeneration following 45 minutes of irradiation per eye. The results obtained in this study could help us better understand the dose-dependent effects of RP-101074, and its potential utility in clinical settings.

**Figure 3:**
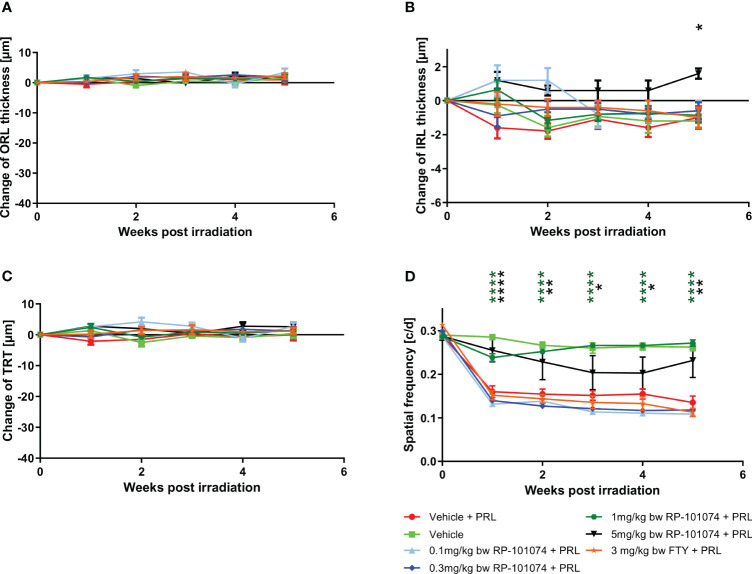
Dose finding and comparative study. **(A-C)** Structural analysis of the outer retinal layers **(A)**, inner retinal layers **(B)**, and total retinal thickness **(C)** of C57BL/6J mice over five weeks after LWI-LI-PRL. **(D)** Visual function measurement by spatial frequency. All graphs represent the pooled mean ± SEM (n=5 animals (10 eyes) per group in total, in two independent experiments), with *p<0.05, **p<0.01, ****p<0.0001 compared to the vehicle + PRL group (if not otherwise mentioned) by GEE analysis. Multiple comparison: Dark green asterisk=1 mg/kg BW RP-101074 group vs. Vehicle + PRL group. Black asterisk=5 mg/kg BW RP-101074 group vs. Vehicle + PRL group.

Under weak degenerative conditions, specifically LWI-LI-PRL (10 mm distance from eye to light source), no statistically significant changes were observed in the retinal layer’s structural analysis by OCT (**Figures 3A–C**) in all treatment groups, including vehicle treatment. On the functional level, prophylactic treatment with 1 mg/kg and 5 mg/kg body weight (BW) RP-101074 provided robust protective effects, preventing visual function loss after LWI-LI-PRL. These effects followed a bell-shaped dose-response curve, as the 1 mg/kg (Week 5: P=<0.0001, MD=0.1365) dose was more effective than the 5 mg/kg dose (Week 5: P=0.0066, MD=0.09651). Other doses did not lead to improved visual acuity (**Figure 3D**).

### 3.2 Retinal structural analysis and visual function analysis after HHI-LI-PRL treatment (strong neurodegenerative conditions)

After identifying the most effective dose of 1 mg/kg BW of RP-101074 for improving visual function in LWI conditions, the mice’s eyes were irradiated with high-intensity light, here, HHI-LI-PRL (5 mm distance from eye to light source), to analyze the effect of 1 mg/kg BW RP-101074 in strong neurodegenerative conditions.

**Figure 4:**
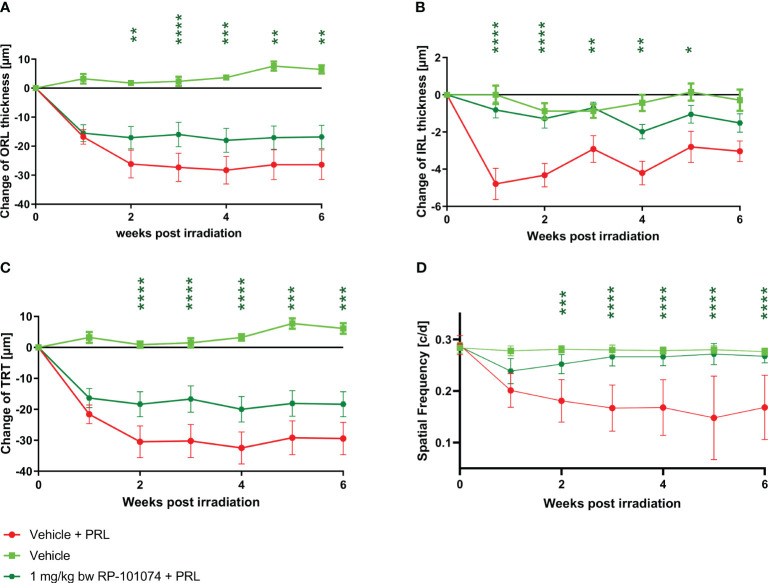
Retinal structural analysis and visual function analysis after HHI-LI-PRL treatment. Structural analysis of the outer retinal layers **(A)**, inner retinal layers **(B)**, and total retinal thickness **(C)** of CX3CR1GFP mice over 6 weeks after HHI-LI-PRL. **(D)** Visual function by spatial frequency. All graphs represent the pooled mean ± SEM (n=6 animals (12 eyes) per group in total, in two independent experiments), with *p<0.05, **p<0.01, ***p<0.001, ****p<0.0001 compared to the vehicle + PRL group (if not otherwise mentioned) by GEE analysis. Multiple comparison: Dark green asterisk=1 mg/kg BW RP-101074 group vs. Vehicle + PRL group.

The structural analysis of the retinal layers and visual function measurement after HHI-LI-PRL revealed beneficial effects of RP-101074 when applied at a dose of 1 mg/kg BW (**Figures 4–C**). A prominent degeneration of all retinal layers occurred already 1 week after irradiation. The neurodegenerative process was significantly reduced when treating the animals prophylactically with 1 mg/kg BW of RP-101074, starting on the day of irradiation. The reduction of the IRL thickness (B) (Week 1: P=<0.0001, MD=-3.971) reached its maximum already 1 week after irradiation, followed by a recovery phase, while total retinal (C) (Week 4: P=<0.0001, MD=-12.50) and ORL (A) (Week 4: P=<0,0004, MD=-12.28) thickness showed increasing degeneration until week 4. Even 6 weeks after irradiation, the effect of RP-101074 regarding TRT and ORT change was still significant compared to untreated control mice (Week 6 ORL: P=<0.0011, MD=-9.576), (Week 6 TRT: P=<0.0003, MD=-11.09).

The visual function was reduced after HHI-LI-PRL treatment, initially in both the RP-101074 treated (Week 2: P=<0.0001, MD=-0.07077) and the untreated group (Week 2: P=<0.0001, MD=-0.09965). However, after 3 weeks, the visual function was restored almost completely in the RP-101074 treated group (Week 3: Mean=0.2787 (Vehicle control) vs. Mean=0.2654 (treated)), while the vehicle + PRL group showed continuous loss of function until week six compared to vehicle control (Week 6: P=<0.0001, MD=-0.1066).

### 3.3 Effect of RP-101074 on microglial infiltration in the CNS of CX3CR1^GFP^ mice after HHI-LI-PRL

To study the effects of 1 mg/kg BW RP-101074 on myeloid cells after HHI-LI-PRL irradiation, confocal scanning laser ophthalmoscopy (cSLO) was used to investigate the infiltration of GFP expressing myeloid cells in the CNS, specifically in the retina around the optic disc, using the CX3CR1-GFP transgenic mouse line, which expresses GFP under the CX3C chemokine receptor 1 (CX3CR1) promotor.

**Figure 5:**
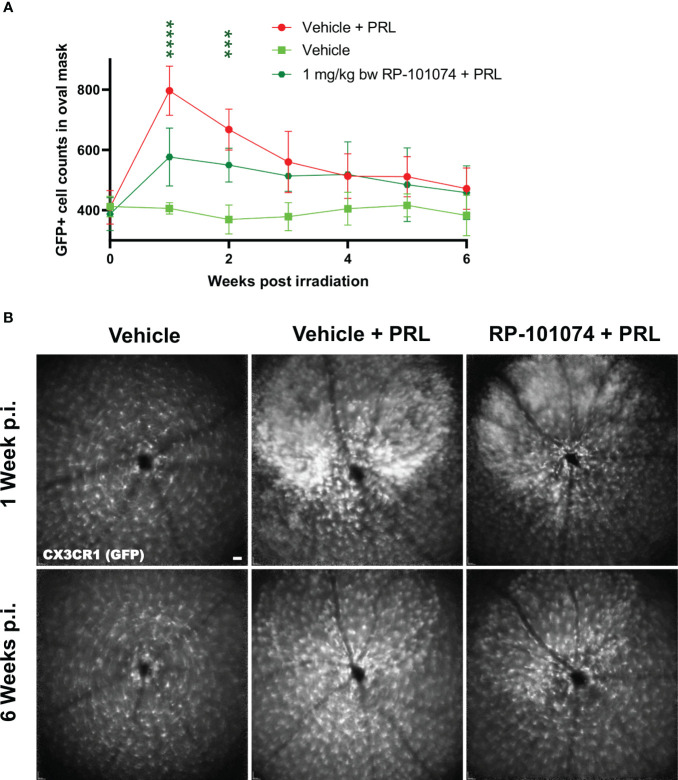
GFP+ cell counts in the retina of CX3CR1^GFP^ mice after HHI-LI-PRL treatment. **Figure 5** demonstrates automated GFP+ cell counts in the retina of CX3CR1GFP mice after HHI-LI-PRL treatment as shown in **Figure 2**. The results of the cell count are presented in **(A)**, with the process repeated for all captures over a period of 6 weeks post-irradiation (p.i.). **(B)** shows example images one week p.i. and 6 weeks p.i. Scale-bar: 200 µm. All graphs show the pooled mean ± SEM (n=6 animals (12 eyes) per group in total, in two independent experiments), with ***p<0.001, ****p<0.0001 when compared to the vehicle + PRL group (if not otherwise mentioned) using GEE analysis. Multiple comparison: Dark green asterisk=1 mg/kg BW RP-101074 group vs. Vehicle + PRL group.

As shown in **Figure 5**, a significant infiltration of myeloid cells in the CNS was observed 1 week after HHI-LI-PRL (Mean=796.9) compared (P=<0.0001) to vehicle control (Mean=402.1), while the infiltration was significantly reduced in the RP-101074 treated group (Mean=576.6, P=<0.0001). At week 4 post irradiation and henceforth, the number of myeloid cells in the CNS was similar in the vehicle + PRL and RP-101074 + PRL group, converging to the cell number counted at the baseline measurement before HHI-LI-PRL treatment.

### 3.4 Histological analyzes of optic nerve longitudinal sections after HHI-LI-PRL

To address the question whether HHI-LI-PRL treatment impacts myelination status and whether RP-101074 exhibits a prophylactic effect, longitudinal sections of optic nerves were subjected to immunohistochemical staining for myelin basic protein (MBP) and Sex determining region Y (SRY) - box 2 (Sox2). MBP serves as a myelin marker, while Sox2 is a marker for neural stem cells and newly differentiated myelinating oligodendrocytes (mOLs).

**Figure 6:**
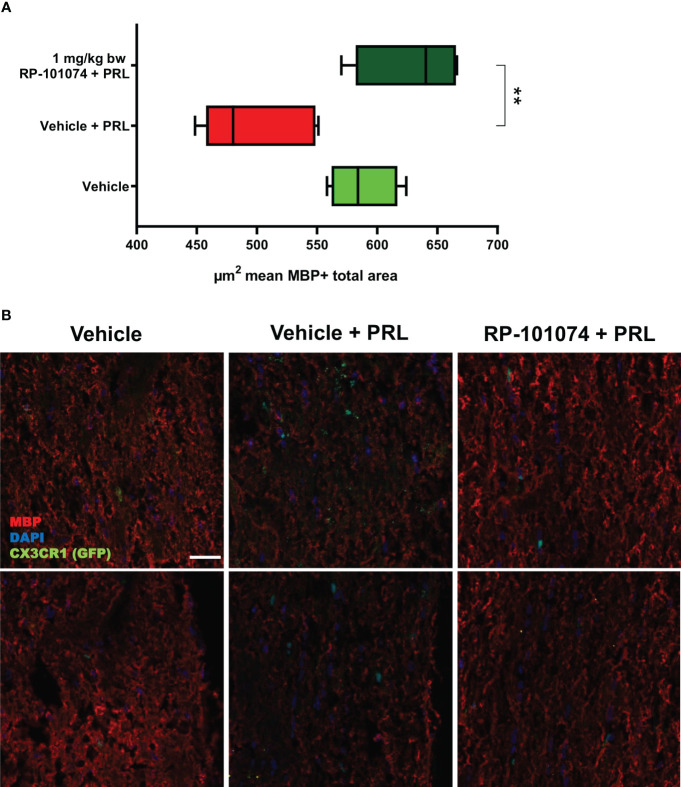
MBP+ total area measurements in optic nerves of CX3CR1^GFP^ mice: **Figure 6** illustrates the automated total area measurement for MBP staining in longitudinal optic nerve sections of CX3CR1-GFP mice as shown in **Figure 2**. The results are shown in **(A)**. Representative images **(B)** portray the longitudinal optic nerve sections with MBP staining (red). The indicated µm^2^ mean of MBP+ total area has been calculated out of a total 8.100 µm^2^ captured area. All graphs depict the pooled mean ± SEM (n=6 animals (12 eyes) per group in total, in two independent experiments), with **p<0.01, as determined by one-way ANOVA with Dunnett’s *post hoc* test.

As evidenced in **Figure 6**, HHI-LI-PRL led to a decrease in MBP-positive myelin within the optic nerve sections (Mean=498.6). In contrast (P=<0.0018), prophylactic treatment with RP-101074 significantly increased the amount of myelin (Mean= (Mean=629.3), suggesting its protective role in maintaining myelination status.

**Figure 7:**
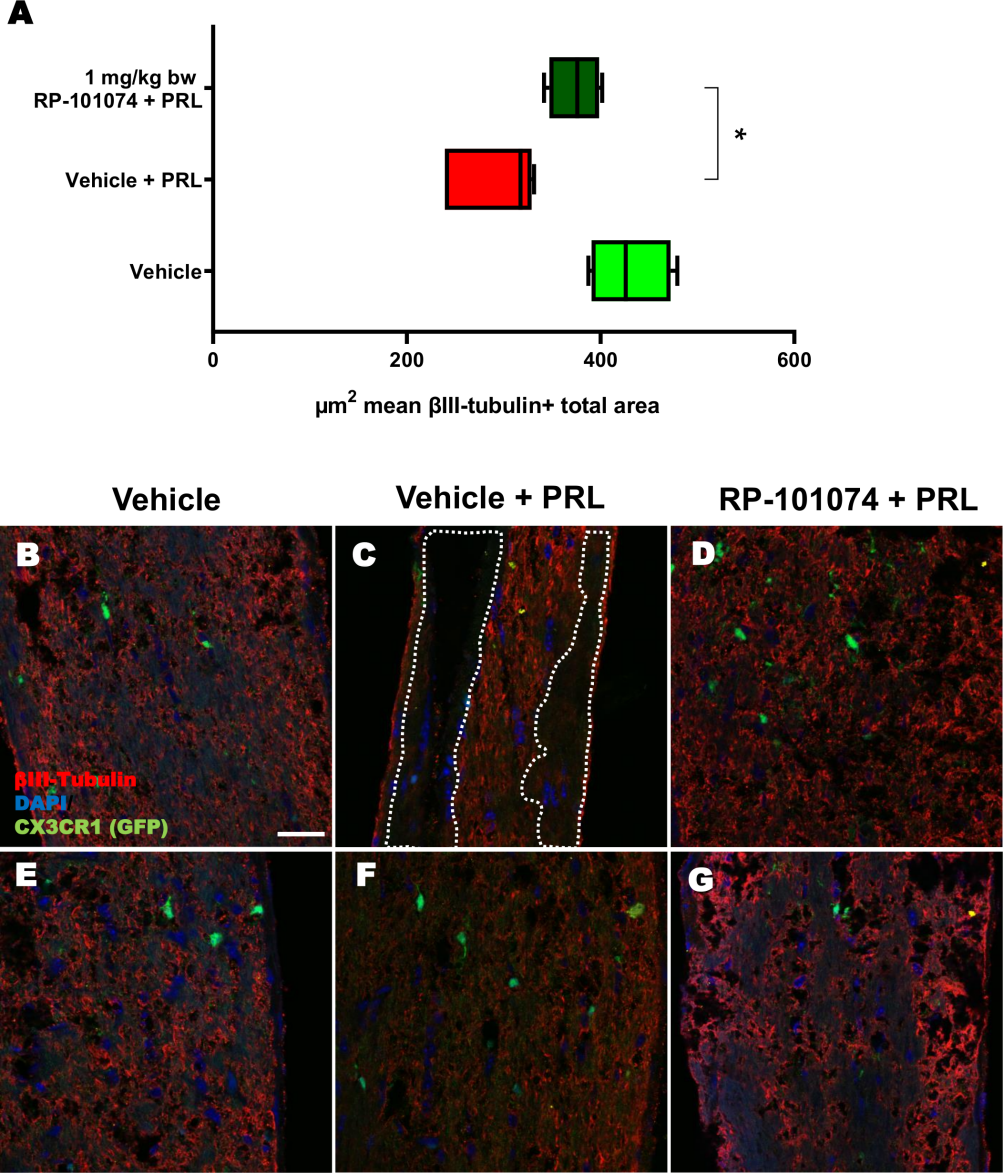
βIII-Tubulin+ total area measurements in optic nerves of CX3CR1^GFP^ mice. **Figure 7** presents the outcomes of an automated total area measurement for βIII-Tubulin staining in longitudinal optic nerve sections of CX3CR1GFP mice as shown in **Figure 2**. The results are illustrated in Chart **(A)**, while Figures **(B–G)** displays representative images. The marked area within the example image **(C)** denotes regions with axonal damage. The indicated µm^2^ mean of βIII-Tubulin+ total area has been calculated out of a total 8.100 µm^2^ captured area. All graphs represent the pooled mean ± SEM (n=6 animals (12 eyes) per group in total, in two independent experiments) with *p<0.05, determined by 1-way ANOVA with the Dunnett *post hoc* test.

HHI-LI-PRL administration led to a decrease in βIII-Tubulin-positive axons within the optic nerves. The representative images of optic nerves post-HHI-LI-PRL treatment depict instances with completely damaged axonal structures (**Figure 7C**) and others with milder axonal damage, as evidenced by reduced βIII-Tubulin expression (**Figure 7F**). Prophylactic RP-101074 treatment, initiated on the day of irradiation, resulted in diminished axonal damage, as demonstrated by increased (Mean=429.8) βIII-Tubulin abundance in the optic nerves (**Figures 7D, G**) in comparison (P=<0.0247) to the vehicle-treated group (Mean=290.7) (**Figures 7B, E**).

**Figure 8:**
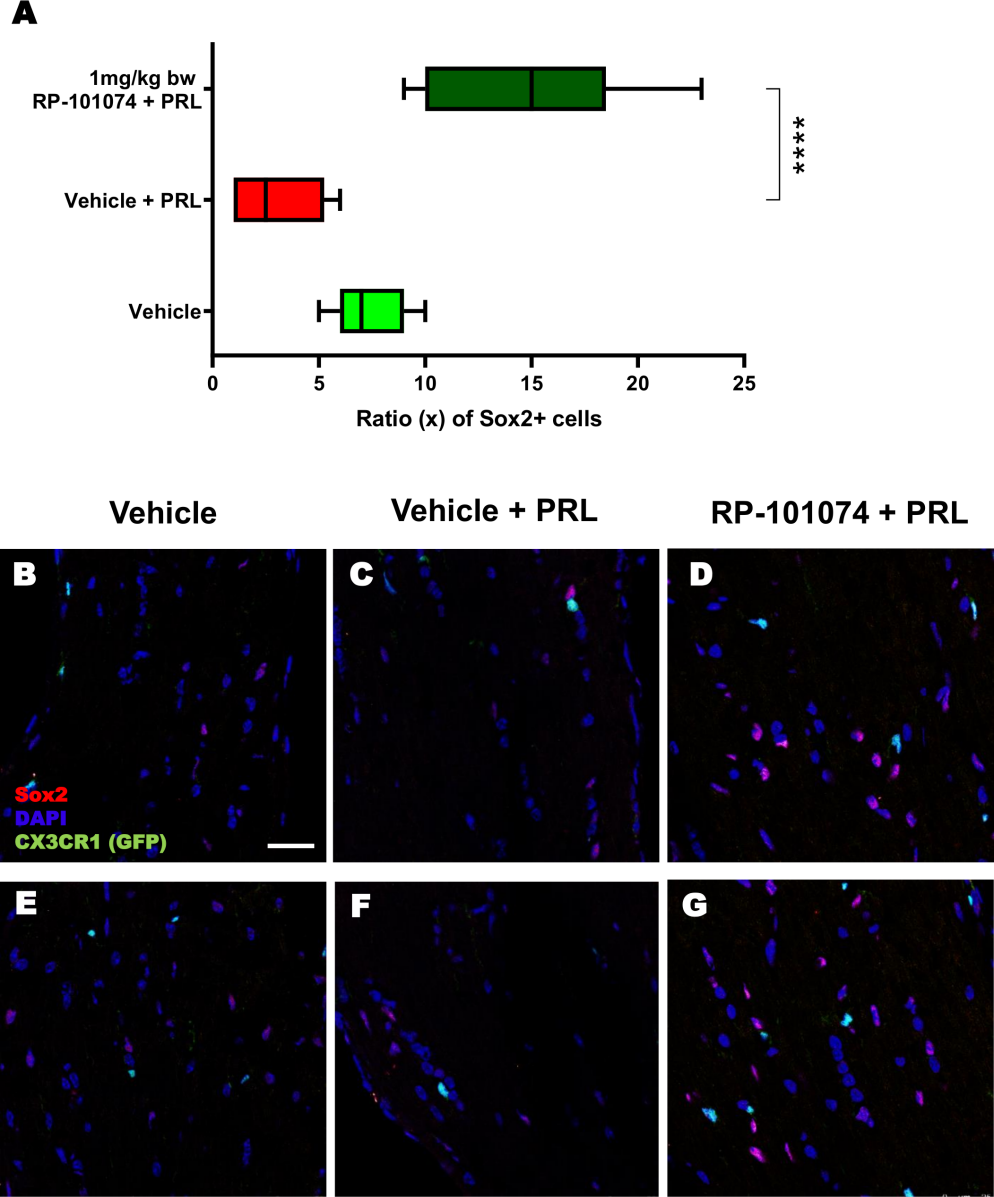
Sox2+ cell counts in optic nerves of CX3CR1^GFP^ mice. Cell counting of Sox2+ cells in relation to the total cell number (DAPI) was performed in longitudinal optic nerve sections of CX3CR1-GFP mice. Figures **(B–G)** exhibit example images, while the results are presented in Figure **(A)**. X-axis of **(A)** indicates the ratio (x) of Sox2+ cells to total cells (DAPI) in % (x/100). All graphs represent the pooled mean ± SEM (n=6 animals (12 eyes) per group in total, in two independent experiments), with ****p<0.0001 determined by 1-way ANOVA with the Dunnett *post hoc* test.

The outcomes displayed in **Figure 8** (Sox2 expression) follow a similar pattern to those of MBP expression (**Figure 6**). HHI-LI-PRL treatment led to a reduced number of Sox2-positive neural stem cells (Mean=3.1). Prophylactic RP-101074 treatment (Mean=14.67) resulted in a significant (P=<0.0001) increase in Sox2-positive cell numbers within the HHI-LI-PRL model.

**Figure 9:**
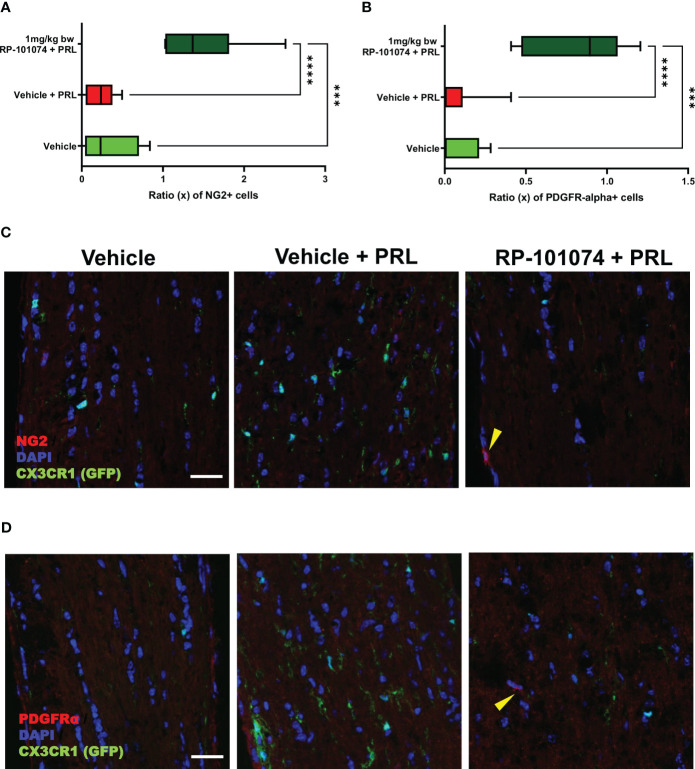
NG2 and PDGFRα expression in optic nerves following photoreceptor loss. Expression levels of OPC markers, NG2 and PDGFRα, were assessed in longitudinal optic nerve sections. Figures **(C, D)** provide representative images for NG2 and PDGFRα respectively, while the quantitative results are portrayed in Figures **(A, B)**. Quantification was accomplished using ImageJ by blinded raters. X-axis of Figures **(A, B)** represent the relative expression levels (% of DAPI cells) of NG2 and PDGFRα, respectively. Yellow arrows indicates positive counted cells. All graphs represent the pooled mean ± SEM (n=6 animals (12 eyes) per group in total, in two independent experiments), with ***p<0.001, ****p<0.0001 determined by 1-way ANOVA with the Dunnett *post hoc* test.

To further elucidate the potential protective impact of RP-101074, we evaluated its influence on oligodendrocyte progenitor cell (OPC) markers, NG2 and PDGFRα. Longitudinal optic nerve sections were subjected to immunohistochemical staining for these markers. **Figure 9A** reveals the relative expression of NG2 in the different experimental groups. Remarkably, we observed a significant upregulation of NG2 in the RP-101074-treated group (Mean=1.481), compared to both the vehicle group (P=0.0005, Mean=0.325) and the HHI-LI-PRL group (P=<0.0001, Mean=0.239). This indicates a potential role of RP-101074 in promoting OPC proliferation or survival following PRL treatment. The representative images of NG2 immunostaining across the experimental groups are illustrated in **Figure 9C**.

The effects of RP-101074 treatment on PDGFRα expression, another OPC marker, are depicted in **Figure 9B**. Consistent with the NG2 expression trend, there was a notable increase in PDGFRα levels in the RP-101074-treated group (Mean=0.819) compared to both the vehicle group (P=0.0001, Mean=0.071) and the HHI-LI-PRL group (P=<0.0001, Mean=0.069). This finding further corroborates the proposed protective and promyelinating role of RP-101074 in the context of PRL. Representative images of PDGFRα immunostaining in the different experimental conditions are shown in **Figure 9D**.

Collectively, these findings suggest that RP-101074 treatment leads to a significant upregulation of OPC markers, NG2 and PDGFRα, pointing towards a beneficial effect in enhancing oligodendrocyte progenitor populations following PRL.

## 4 Discussion

In this study, the protective capacity of the S1PR-1/-5 modulator RP-101074 was assessed in a primary degenerative model of photoreceptor degeneration induced by light overstimulation. Initially, we established a standardized protocol wherein all mice were subjected to identical conditions during LI-PRL treatment, maintaining consistent parameters such as the angle of the light source to the eye, light intensity, light temperature, and light exposure duration. Given that the *in vivo* OCT and cSLO measurements were performed around the optic disc in a specified area, the eye/head position of the mice during LI-PRL treatment was standardized to ensure that the primary focus of the light beam targeted the same retinal area in all mice. One of the major factors influencing the degree of degeneration in photoreceptors and the retina following LI-PRL is the distance between the light source and the eye. In our dose-finding study, we initially applied low-intensity (LWI) LI-PRL to induce mild retinal degeneration in the animals’ retinae. However, while OMR revealed reduced visual acuity, no significant degeneration was observed in the retinal layer’s structural analysis using OCT among all groups. An intermediate RP-101074 concentration (1 mg/kg BW) provided more pronounced protection from visual function loss after LWI-LI-PRL than a higher concentration of 5 mg/kg BW, resembling a bell-shaped dose-response curve. This finding was consistent with our previous studies using the S1PR 1-/-5 modulator siponimod, wherein the lower dose was more effective than the higher dose in preventing retinal neurodegeneration during experimental optic neuritis (15). Recent studies conducted by peers researching S1PR modulators also exhibit bell-curve dose-dependent results (18, 19). A plausible explanation is that a high dose of RP-101074 (5 mg/kg BW) leads to a more potent internalization and degradation of S1P receptors (compared to 1 mg/kg BW), resulting not only in immune-modulating effects primarily attributed to the modulation of S1PR-1 but also in cascades that influence the function of oligodendrocytes and other cells in the CNS expressing S1PR-5 (20). The observed bell-shaped dose-response curve corresponds with earlier studies on FTY720, another S1P receptor modulator, further highlighting the intricate and often counterintuitive pharmacodynamics of these compounds (21).

After observing limited discernible outcomes after mild irradiating conditions (LWI), we decided to explore more potent conditions to induce degeneration to the retinal tissue. We here only employed the most efficacious dose of 1 mg/kg in the (HHI) setting due to our observation, that doses lower or higher doses failed to elicit significant effects under LWI. We concluded, that other doses would likely not yield substantial effects under the stronger HHI conditions, as they did not exhibit any noticeable impact even under the comparatively milder LWI setting. Although the protective effects of 5 mg/kg BW RP-101074 were also significant, we opted to use the most effective dose of 1 mg/kg BW RP-101074 in our subsequent HHI LI-PRL studies. A recent study showed, that ozanimod showed efficacy at doses of 0.2 mg/kg in a mouse model of experimental autoimmune encephalomyelitis. This dose is 5 times lower than the dose of 1mg/kg RP-101074, which was proved most effective in our study. Due to their calculations, the predicted human equivalent dose would be 0.96 mg, very similar to the 0.92 mg label dose of ozanimod (22). However, it is important to note that the model used in the cited work differs from our model, particularly regarding intensity of retinal inflammation. Doses of 0.1 and 0.3 mg per day used in our study did not yield in any beneficial effects in the LWI setting. We, therefore, hypothesize that the 0.2 mg dose would also not yield significant effects under HHI radiation. However, we acknowledge that it cannot completely excluded that doses lower that 1 mg could have beneficial effects under the HHI conditions. It is important to highlight that the substance utilized in our study was not ozanimod but rather RP-101074 and that no comparable dose finding data are available.

As illustrated in **Figure 4**, prophylactic RP-101074 treatment resulted in significant protection from retinal degeneration in both inner and outer layers. As depicted in **Figure 4A**, one week after HHI-LI-PRL, the degeneration of the outer retinal layers, including the photoreceptor layer, was similar in both irradiated groups (vehicle + PRL and RP-101074 + PRL). This observation suggests that the photoreceptor layer predominantly degenerates in the context of HHI-LI-PRL. Interestingly, the degeneration of the inner retinal layers, which encompass the retinal nerve fiber layer, ganglion cell layer, and their dendrites in the inner plexiform layer, was only significantly induced after HHI-LI-PRL in the group without RP-101074 treatment. This finding indicates that RP-101074 appears to exhibit protective effects on both directly affected photoreceptor cells and indirectly on ganglion cells and their axons. These protective effects were observed in both short-term (**Figure 4A**, two weeks after HHI-LI-PRL treatment) and long-term follow-ups (**Figure 4A**, six weeks after HHI-LI-PRL treatment). The increased abundance of Sox2-positive neural stem cells upon RP-101074 treatment, as shown in **Figure 8**, suggests that the protective action of this treatment may involve the activation and/or recruitment of neural progenitors and/or the release of neuroprotective factors, in addition to the modulation of inflammatory responses. The activation and recruitment of neural progenitors as a response to RP-101074 treatment aligns with findings from 23, where modulation of S1P receptors was found to enhance the regenerative capacity of neural cells in a murine stroke model, further hinting at the multi-faceted therapeutic potential of such modulators.

As described above, the change in inner retinal thickness (indirectly affected cells) seems to occur at an earlier time point, specifically, one week after HHI-LI-PRL, than the changes in the outer retinal layers (directly affected cells), where the most pronounced degeneration was observed two weeks after HHI-LI-PRL (**Figure 4**). This finding might be attributable to swelling of the outer retinal layers following inflammatory processes, where microglial migration from the inner retinal layers to the outer retinal layers, as well as an amoeba-like shift of microglial cells, occurs during inflammation (24).

Microglial activation has been linked to retinal degeneration in CNS disorders (25) and others like Alzheimer’s, Parkinson’s, and Huntington’s diseases (26); therefore, the number of CX3CR1-positive microglia was evaluated *in vivo* using cSLO over a period of six weeks following HHI-LI-PRL. As depicted in **Figure 5**, RP-101074 treatment resulted in significantly reduced numbers of CX3CR1-positive cells around the optic disc in the retina one week after HHI-LI-PRL, suggesting a diminished activation of the innate immune response in the retina. This effect can likely be attributed to the immunomodulatory mode of action of S1PR-1/-5 modulators (15, 20). This effect was most prominent one week after irradiation and was followed by a convergence of microglial cell counts between RP-101074- and vehicle-treated groups within the subsequent five weeks. Our own and other studies have even suggested a shift of microglial cells under S1PR-modulator treatment towards a regenerative phenotype (15, 27). Despite observing most significant changes within the first 3 weeks after irradiation, we made the decision to extend the observational period in order to thoroughly evaluate the progression of neurodegeneration and potential regenerative processes following the resolution of inflammation, also focusing on potential regenerative aspects of the treatment (28).

Interestingly, Naruse and colleagues found in 2018, that microglial activation could induce OPC generation following focal demyelination, suggesting a potential role for these cells in regeneration (29). Other recent studies show that S1PR modulators shows growing evidence that targeting S1PR modulates mechanisms beyond immunomodulation, such as remyelination (15, 30, 31). Furthermore, S1PRs could also play a direct role in the regulation of stem cell migration, proliferation, and differentiation (32). Understanding the details between S1PRs and its pathway contributing to shifting myeloid activity or even direct stem cell interaction will be crucial in developing novel therapeutic strategies to modulate the behavior of these versatile cells in neurodegenerative diseases.

Axonal damage and demyelination resulting from autoimmune inflammation are among the main reasons for clinical disability in MS patients and their animal models. Consequently, we further investigated whether RP-101074 affected the myelin status and axonal integrity of the optic nerve in our model. We analyzed longitudinal sections of the optic nerves, staining for MBP, a marker for myelin, and Sox2, a marker for neural stem cells and for newly differentiated myelinating oligodendrocytes (mOLs) in the adult brain and spinal cord. MBP is a sensitive marker of myelination (33), indicating the involvement of differentiated, myelinating oligodendrocytes. Sox2, on the other hand, is one of various transcription factors critical for oligodendrocyte (OL) development, OL regeneration, and OPC (oligodendrocyte progenitor cell) differentiation (34). Recent studies suggest that Sox2 may act as a transcriptional master regulator at the epigenetic level, affecting the expression of crucial downstream factors involved in oligodendrocyte development (35).

Histological analyses of longitudinal sections of the optic nerve demonstrated significantly decreased myelination and reduced neural stem cell numbers following HHI-LI-PRL. Prophylactic treatment with RP-101074 resulted in significantly increased numbers of Sox2+ cells compared to the vehicle control group and a higher amount of myelin compared to the vehicle + PRL group. Furthermore, elevated expression levels of NG2 and PDGFRα in the RP-101074 treated group not only align with the observed increase in myelination and progenitor cell numbers but also hint towards a possible role in fostering OPC populations. We, therefore, conclude that RP-101074 treatment might lead to the recruitment of OPCs and/or regeneration of OLs, resulting in increased remyelination. To further assess whether the reduced amounts of myelin after HHI-LI-PRL were due to axonal damage, βIII-Tubulin staining of longitudinal optic nerve sections was performed. The analysis revealed prominent axonal damage in the context of HHI-LI-PRL, while animals prophylactically treated with RP-101074 displayed higher amounts of βIII-Tubulin, indicating protective effects. These effects may, at least in part, explain the increased myelin abundance in the optic nerve upon RP-101074 treatment during HHI-LI-PRL.

The reduction of retinal degeneration observed by OCT in the RP-101074 group is certainly at least in part resulting from immunomodulation, including decreased microglial activation or recruitment. Nevertheless, our histological evaluations of myelination (MBP), progenitor cells (Sox2) and OPC´s (NG2, PDGFRα) demonstrate an increased expression in the RP-101074 treated animals even when compared to the untreated control group. These observations suggest an impact of RP-101074 that is not solely due to its immunomodulatory function. While it is possible that our data predominantly exhibits an anti-inflammatory effect, the increased myelination and number of progenitor cells and OPC´s in the RP-101074 group could suggest an additional protective or regenerative mode of action.

Nevertheless, our research is not without limitations. While the presented light-induced photoreceptor loss is a model for mimicking neurodegeneration without a T- and B-cell driven component, it still includes inherent inflammatory effects. As such, it remains unclear if the observed effects of RP-101074 are primarily due to its effects on OPCs and myelin or the result of reduced microglial response leading to less neuronal damage. Additionally, our LI-PRL model, although successful in representing certain elements of neurodegeneration, might not fully grasp the multifaceted pathophysiology of human diseases involving neurodegeneration. Hence, validating the applicability of our findings to additional preclinical models and of course human pathology is essential. It would be beneficial to further examine the RP-101074’s anti-inflammatory and neuroprotective properties in detailed mechanistic studies involving a variety of preclinical models to better understand its therapeutic potential.To summarize, our preliminary research suggests that RP-101074, an S1PR-1/-5 modulator, might have beneficial effects that extend beyond its known immunomodulatory action. This was demonstrated using the LI-PRL primary degenerative model. The protective effects could be potentially linked to the enhanced recruitment of Sox2+ cells and oligodendroglial cells, along with the regulation of microglial cell activity via S1PR-1.

## Data availability statement

The original contributions presented in the study are included in the article/supplementary material. Further inquiries can be directed to the corresponding author.

## Ethics statement

The animal study was reviewed and approved by State Agency for Nature, Environment and Consumer Protection; AZ 81-02.04.2019.A063.

## Author contributions

MS, CH, AI and MD performed the experiments and analyzed the data. MS, MD and PA wrote the manuscript. SM, TR, PA and MD were involved in revising the manuscript critically for important intellectual content and made substantial contributions to interpretation of data. PA and MD conceived the study and supervised experiments. All authors contributed to the article and approved the submitted version.
